# Stroke detection in medical emergency calls: a retrospective analysis and exploratory evaluation of an AI decision support model

**DOI:** 10.1186/s13049-026-01645-x

**Published:** 2026-06-09

**Authors:** Emil Iversen, Hege Ihle-Hansen, Kari Krizak Halle, Alexander Selvikvåg Lundervold, Lars Myrmel, Anders Strand Vestbø, Annette Fromm, Cosimo Damiano Persia, Christian Autenried, Guttorm Brattebø

**Affiliations:** 1 Norwegian National Advisory Unit on Emergency Medical Communication, Haukeland University Hospital, Bergen, Norway; 2 Oslo Emergency Medical Communication Centre, Pre-hospital Clinic, Oslo University Hospital, Oslo, Norway; 3 Department of Medicine, Vestre Viken Hospital Trust, Drammen, Norway; 4 Department of Medical Quality Registries, St Olavs Hospital, Trondheim University Hospital, Trondheim, Norway; 5 Department of Strategy and Management, NHH Norwegian School of Economics, Bergen, Norway; 6 Department of Radiology, Haukeland University Hospital, Bergen, Norway; 7 Department of Anaesthesia & Intensive Care, Haukeland University Hospital, Bergen, Norway; 8 Department of Research and Development, Haukeland University Hospital, Bergen, Norway; 9 Department of Neurology, Haukeland University Hospital, Bergen, Norway; 10 Team Artificial Intelligence, Helse Vest ICT, Bergen, Norway; 11 Department of Clinical Medicine, University of Bergen, Bergen, Norway; 12 Department of Nursing and Health Promotion, Faculty of Health Sciences, Oslo Metropolitan University, Oslo, Norway

**Keywords:** Artificial intelligence, Decision support, Emergency medical communication centre, Emergency medical dispatch, Emergency medical services, Medical emergency calls, Stroke

## Abstract

**Background:**

Timely identification of acute stroke during medical emergency calls is critical for optimizing patient outcomes. We aimed to (i) characterize the prehospital trajectories of patients with stroke and (ii) explore the ability of an artificial intelligence (AI) model developed within the Artificial Intelligence Support in Medical Emergency Calls project to support decision-making at Emergency Medical Communication Centres (EMCCs).

**Methods:**

We conducted a retrospective analysis using data from 1,164 patients diagnosed with stroke from an EMCC (2018–2019), focusing on those primarily assessed by EMCC operators. We categorized patients into optimal and nonoptimal trajectory groups and applied logistic regression to explore the factors associated with an optimal trajectory. An AI model was trained using a dataset of 2,980 emergency calls (2019 and 2022), integrating transcribed audio logs and structured clinical data. The model was evaluated on the full dataset and subgroups that were incorrectly assessed by the EMCC.

**Results:**

Aphasia/dysarthria was the only factor associated with optimal trajectories. The AI model achieved a sensitivity of 81.0%, specificity of 79.6%, and F1-score of 64.7%. The subgroup analysis included 14 false-negative and 41 false-positive cases. The model correctly predicted stroke in 9 of 14 false-negative cases and ruled out stroke in 9 of 41 false-positive cases.

**Conclusions:**

This explorative evaluation indicates that a multimodal AI pipeline combining audio and structured clinical data may show potential to support decision making in medical emergency calls. Internal results are promising, but studies on larger and external datasets are needed to validate these findings.

**Graphical Abstract:**

**Figure:**
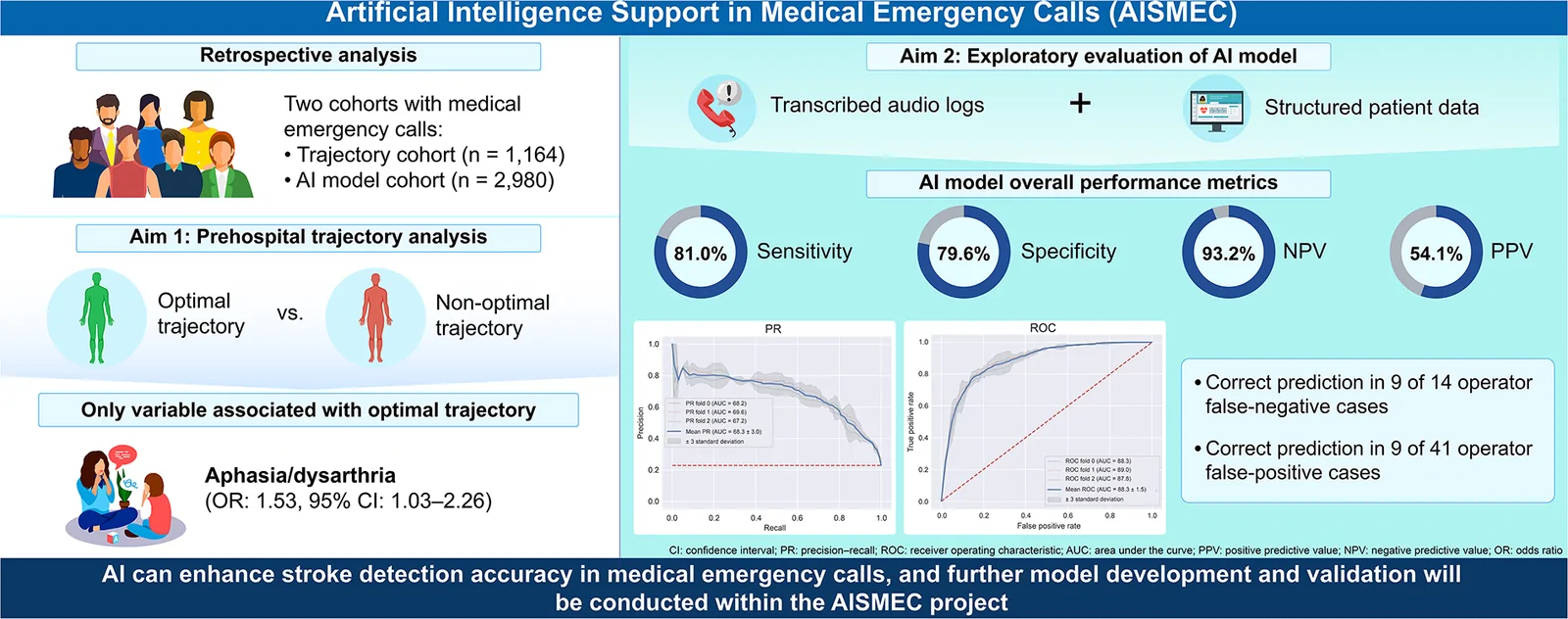

**Supplementary Information:**

The online version contains supplementary material available at 10.1186/s13049-026-01645-x.

## Background

### Introduction

Early identification of patients with acute stroke during medical emergency calls is essential for effective treatment [1–4]. Correct decision-making by the Emergency Medical Communication Centre (EMCC) operators is associated with reduced prehospital delays and faster in-hospital care [3–7]. In Norway, EMCCs use the Norwegian Index for Emergency Medical Assistance (Index) to triage inquiries as “acute” (ambulance with lights and sirens), “urgent” (ambulance dispatched or call transferred to an out-of-hours primary care call centre), or “regular” (non-urgent) (Additional file 1) [8]. To determine the urgency, the operator selects a relevant page in the Index, for example “Stroke symptoms,” based on the presented complaint and checks for symptoms that match the criteria listed on that page. The Index is a pdf-based criteria-based decision support tool with a limited sensitivity and positive predictive value, but good negative predictive value and specificity [9]. Further decision support is not readily available for the EMCC operators.

### Importance

There is a strong need to improve prehospital acute stroke care to optimize patient outcomes [3, 10–12]. Current decision support for EMCC operators is limited and relies primarily on criteria- or algorithm-based systems, and prior medical history is not readily available. The introduction of artificial intelligence (AI) as an additional decision support in the EMCC workflow may help address these limitations and may hold promise for improving stroke detection accuracy [13–15]. A Danish study suggested that an AI model analysing words from medical emergency calls could enhance stroke detection [16]. We believe that adding further structured clinical data to an AI model based on word analysis may improve accuracy. Effective implementation of AI in the EMCC requires a deeper understanding of the “Stroke Chain of Survival” [17]. Few studies have examined stroke pathways in this phase, and to our knowledge, none have explored factors influencing these pathways [10, 12, 18]. Importantly, there is limited existing evidence on AI approaches combining analysis of emergency call audio with structured clinical data, suggesting a gap in the current evidence base.

### Goals of this investigation

The primary objective of this study was to conduct an exploratory evaluation of the AI Support in Medical Emergency Calls (AISMEC) AI model using the full dataset and within subgroups of EMCC stroke false negatives (“stroke chameleons”) and false positives (“stroke mimics”). Secondary objectives were to characterize patients with acute stroke, analyse their trajectories through the emergency medical communication system, and explore factors associated with optimal handling by the EMCC, thereby providing background and context for the development of the AI model.

## Methods

### Study design

This is a retrospective, registry-based study.

### Setting

This study was conducted within the catchment area of Bergen EMCC. Bergen EMCC, in the Western part of Norway, serves a population of approximately 470,000 inhabitants and handles ~ 56,000 medical emergency calls annually. All hospital admissions were to one of the following hospitals in the region: Haukeland University Hospital, Haraldsplass Deaconess Hospital, or Voss Hospital.

### Selection of participants

We classified patients into the trajectory and AI model cohorts.

#### Trajectory cohort

The trajectory cohort comprises 1,164 patients diagnosed with acute stroke, identified through the Norwegian Stroke Registry (NSR). Inclusion in the NSR requires hospital admission with an ICD-10 stroke diagnosis (I61, I63, or I64). All patients suffered a stroke between January 1, 2018, and December 31, 2019. For the study of stroke patient trajectories, we included only patients primarily evaluated by the EMCC; thus, those evaluated by a doctor before EMCC contact were not included in further analyses. These cases were excluded because they represent a different clinical pathway in which stroke suspicion may already have been established before EMCC involvement, and their inclusion could therefore bias the assessment of EMCC triage and decision-making.

Registry data from the mandatory NSR on reported symptoms and other patient- and stroke- related variables (Additional file 2) were merged with manually recorded data describing the prehospital trajectory for each patient, from the debut of stroke symptoms to the departure from the scene of the incident. Manually recorded data were obtained from the patient record system in the EMCC, the Emergency Medical Information System (AMIS) (Additional file 3), and emergency call audio logs. As audio logs from 2018 were routinely deleted from the EMCC database after three years of collection, we only had access to audio logs from 2019.

#### AI model cohort

As part of the AISMEC project, we developed an AI model to improve stroke detection accuracy at EMCCs. For model development, we created a dataset of 2980 patients who made emergency calls to the Bergen EMCC in 2019 and 2022. Stroke audio logs from 2019 had already been extracted as part of the AISMEC project, and additional AI model cohort audio logs were from 2022 to ensure that they were not routinely deleted (after 3 years) during the extraction process. The AI model cohort comprised 684 patients with stroke (355 patients from 2019, obtained from NSR, and 329 from 2022, obtained from hospital records) and 2,296 without stroke (2022 only). The no-stroke group was randomly selected as a representative sample of all non-stroke calls to the EMCC in 2022. This approach was intended to represent the broader emergency call population and capture real-world variability in call characteristics. Inquiries related to childbirth, pregnancy, gynaecology, and psychiatry, as well as patients under 18 years of age, were excluded in accordance with recommendations from the Regional Committee for Medical and Health Research Ethics during the process of obtaining ethical approval for the study. All patients had an emergency call audio log, structured clinical data from hospital records, including established stroke risk factors (such as prior stroke or myocardial infarction), and information on pharmacological treatments indicative of elevated stroke risk, including antihypertensive agents, anticoagulants, antidiabetics, and statins. The EMCC system for handling inquiries has remained unchanged from 2018/2019 to 2022.

#### EMCC false-negative and false-positive subgroups

Among patients with stroke in 2019, we manually identified a subgroup of EMCC false-negative cases in which the EMCC did not recognise that the patient was experiencing a stroke during the medical emergency call. In this setting, false negatives were defined as contacts to the EMCC within 4 h of stroke onset, with no indication of EMCC stroke suspicion based on the assigned Index criterion code and a manual review of the operator’s free text in AMIS. In unclear cases, audio logs were additionally reviewed to assess whether stroke suspicion was mentioned. We included an EMCC false-negative subgroup of 14 patients in this study.

An EMCC false-positive subgroup was derived from the 2022 cohort of the AI model cohort. We included patients for whom an acute ambulance dispatch was initiated based on the stroke index criterion code. To ensure that stroke was indeed suspected, a manual review of the operator’s free text in the AMIS was conducted. We included an EMCC false-positive subgroup of 41 cases.

For both subgroups the manual review was restricted to a single reviewer (the first author) to comply with data access limitations imposed by the Regional Committee for Medical and Health Research Ethics. Subgroup analyses of EMCC false-negative and false-positive cases should be considered exploratory.

### Variables

#### Outcome variables

In the trajectory cohort (1,164) from 2018 to 2019, 831 patients were primarily assessed using the EMCC. Based on how these patients were handled by the EMCC, they were assigned to two groups: “optimal course” (669, 81%) or “nonoptimal course” (162, 19%). Patients assigned to the “optimal course” group made direct contact with the EMCC or called via the out-of-hours primary care call centre within 24 h of stroke onset and received an ambulance with acute priority. Patients assigned to the “nonoptimal course” group had either contacted the EMCC within 24 h of stroke onset but were not triaged as “acute” or had contacted the EMCC later than 24 h from stroke onset. To ensure that the patients were assigned to the correct group, we established the variable “time from stroke onset to EMCC contact”. The 24 h cutoff reflects the clinical routines at the Bergen EMCC for acute ambulance dispatch in patients with severe stroke symptoms who contact within 24 h of symptom onset.

#### Variables from national registries

We obtained symptoms and patient- and stroke-related variables from the NSR [19]. The variable “level of consciousness at hospital admission” was divided into two categories: “awake” (0) and “not fully alert” (1), with the latter encompassing all other levels of consciousness. The data of patients with intracerebral haemorrhage were excluded from the analyses of intravenous thrombolysis (IVT) and endovascular thrombectomy (EVT) because these interventions were not indicated for this patient group. The variables “aphasia” and “dysarthria” were combined into a single category, “aphasia/dysarthria,” as outlined in the Index. Consequently, patients may exhibit 0–3 symptoms on the Face-Arm-Speech-Time (FAST) scale. Stroke severity was classified according to The American National Institutes of Health Stroke Scale as very mild (0–2), mild (3–5), moderate (6–14), and severe (15–40) [20].

Data on prescribed medications relevant to stroke risk were obtained from the Norwegian Prescribed Drug Registry (Additional file 4).

#### Variables from hospital records

We retrieved all prehospital contact data and actions registered in the EMCC, along with their corresponding timestamps from the patient record system AMIS. These data encompassed the period from stroke onset until the patient’s departure from the scene of the incident for each case, resulting in a comprehensive chronological timeline for every patient. In instances of uncertainty, we consulted audio logs or operator-written free text for clarification.

Data on prior stroke, myocardial infarction, prior hospital admissions, and other relevant stroke risk factors were obtained from the patient record system at the Distributed Information and Patient Data System (DIPS) (Additional file 5).

### AI model

The AI model was developed using a two-step architecture with separate pipelines tailored to unstructured and structured data sources. Audio recordings from emergency calls were first transcribed using a model based on OpenAI’s Whisper speech-to-text model, which was fine-tuned for medical emergency calls in the AISMEC project [21](see Additional file 6 for details). The resulting transcripts were used as inputs to a text classification model based on ModernBERT [22]. A separate classification model was trained on structured clinical data, comprising historical data from hospital records and information on prescribed medication, using a Random Forest algorithm [23]. The outputs from the two models were subsequently combined to generate a final probability estimate of stroke. Binary classification was based on a probability threshold of 0.5. As the current study represents a preliminary evaluation of the model, no additional threshold optimization was performed. Future work will investigate clinically informed threshold selection strategies, including optimization for sensitivity and specificity in the EMCC setting. Details of the development of the AI model can be found in Additional file 6. Data from hospital records and the Norwegian Prescribed Drug Registry were collected using the national identification number. This number is also collected in medical emergency calls for patient identification, making these data sources available for an AI model in an acute setting also.

### Statistical analyses

For the trajectory cohort, patient characteristics are presented for all patients and those primarily assessed by the EMCC. Missing data were minimal. Descriptive statistics for the two groups are reported as proportions, means, or medians, with interquartile ranges and standard deviations for continuous variables and frequencies, absolute numbers, and proportions for categorical variables.

Multivariable logistic regression was applied to explore associations between experiencing an optimal course and clinically relevant variables. Variables were selected a priori based on clinical relevance. All selected variables were entered simultaneously into the model, and no stepwise selection procedures were used. Separate logistic regression models were fitted for patient- and stroke-related variables and for symptom variables, respectively, with all models adjusted for age and sex. No apparent collinearity between variables was identified based on clinical assessment. To reduce the risk of overfitting, the models were kept parsimonious with a limited number of variables relative to the number of outcome events. Model fit was not formally assessed using goodness-of-fit tests, as the analysis was exploratory in nature and focused on identifying associations rather than predictive performance. The variable “level of consciousness at hospital admission” was excluded from the logistic regression analysis due to potential confounding. Results are presented as odds ratios (ORs) with corresponding 95% confidence intervals (CIs).

The classification performance of the AISMEC AI model was assessed using three-fold stratified cross-validation on the full dataset of 2,980 patients from 2019 and 2022 to maximize the use of available data for both training and evaluation. Results are summarized in a confusion matrix. The model performance was assessed using standard classification metrics, including sensitivity, specificity, positive predictive value (PPV), negative predictive value (NPV), and F1–score, with 95% CIs. The model performance reported reflects internal validation using cross-validation and does not represent external validation.

Given the imbalance in the dataset, with more non-stroke than stroke cases, performance was measured with a precision–recall (PR) curve alongside the receiver operating characteristics (ROC) curve. The PR curve illustrates the trade-off between precision (PPV) and recall (sensitivity).

The model’s performance was further assessed on 14 EMCC false-negative and 41 false-positive cases described in the *Selection of Participants* section. Given the small sample size, this analysis was exploratory and clearly underpowered for reliable AI model performance estimates. Missing data were evaluated prior to analysis. The extent of missing data was low and did not suggest a systematic pattern. Missing numerical values were imputed using the median, and categorical variables were imputed with a constant category (“MISSING”) followed by one-hot encoding. Given the exploratory nature of the study and the limited degree of missingness, no advanced imputation methods were applied. Data were analyzed using IBM SPSS Statistics for Windows, version 29.0.2.0 (IBM Corporation, Armonk, NY, USA). Model development and evaluation were performed using Python version 3.11 (Python Software Foundation, Wilmington, DE, USA) and common scientific computing libraries.

In this study, we followed the Strengthening the Reporting of Observational Studies in Epidemiology (STROBE) guidelines [24]. This study was approved by the Regional Committee for Medical Research Ethics, Western Norway, REK West on March 22, 2024 (REK West No. 2020–108573). The committee granted a waiver of confidentiality obligations, but an information letter was sent to all identified individuals, providing details about the study and offering the opportunity to withdraw at any time. The privacy rights of human subjects were respected throughout the study.

## Results

The dataset included patients with and without stroke in 2018, 2019, and 2022. Of 1,303 patients diagnosed with acute stroke in Bergen in 2018–2019, 1,164 (89%) were included in the trajectory cohort of our study after exclusion due to lack of consent or available AMIS data. Accordingly, we included 831 patients who were primarily assessed by the EMCC for further analysis (Additional file 7).

Among the 2,980 patients used to evaluate the AI model, we manually identified 14 EMCC stroke false negatives from the nonoptimal course group of patients with stroke in 2019. Furthermore, we identified 41 EMCC stroke false positives from the group without stroke in 2022 (Additional file 8).

### Patient characteristics

The 831 patients primarily assessed by the EMCC had a median age of 77 years (Q1–Q3:68–85); 380 (43%) were women. The number of patients in the “optimal course” and “nonoptimal course” groups were 669 (81%) and 162 (19%), respectively (Table 1).

**Table 1:** Trajectory cohort (2018/2019) characteristics

Characteristics	Primary assessment by EMCC (*n* = 831)		All patients (*n* = 1164)
	Optimal course (*n* = 669)	Nonoptimal course (*n* = 162)	Total (*n* = 1,164^*^)
**Age**,** median (Q1–Q3)**	77.0 (68–85)	77.0 (68–85)	77.0 (58–85)
**Sex (female)**,** n (%)**	313 (46.8)	67 (41.4)	545 (46.8)
**Stroke suspected**,** n (%)**	566 (84.6)	71 (43.8)	939 (80.7)
**Wakeup–stroke**,** n (%)**	163(24.4)	36 (22.2)	278 (23.9)
**Type of stroke**,** n (%)**			
Ischemic	581 (86.8)	137 (84.6)	1023 (87.9)
Haemorrhagic	88 (13.2)	25 (15.4)	141 (12.0)
**IVT**,** n (%)**^**†**^	255 (43.9)	19 (13.9)	313 (30.6)
**EVT**,** n (%)**^**†**^	74 (12.7)	4 (2.9)	94 (9.2)
**NIHSS at admission**, **median (Q1–Q3)**	3.0 (1–7)	2.0 (1–5)	3.0 (1–8)
**Stroke severity according to NIHSS**,** n (%)**			
Very mild (0–2)	219 (32.7)	83 (49.1)	485 (41.7)
Mild (3–5)	161 (24.1)	41(25.3)	302 (25.9)
Moderate (6–14)	162 (24.2)	26 (15.4)	226 (19.4)
Major (15–40)	127 (19.0)	11 (6.8)	151 (13.0)
**Level of consciousness (awake)**, **n (%)**	557 (83.3)	149 (92.0)	1018 (87.5)
**NSR symptoms**,** n (%)**			
Leg paresis	344 (51.4)	66 (40.7)	524 (45.0)
Aphasia	281 (42.0)	43 (26.5)	384 (33.0)
Arm paresis	361 (54.0)	72 (44.4)	547 (47.0)
Facial palsy	354 (52.9)	60 (37.7)	528 (45.4)
Aphasia/dysarthria^**‡**^	436 (65.2)	78 (48.1)	653 (56.1)
Other focal total	527 (78.8)	120 (74.1)	899 (77.2)
Vertigo	35 (5.2)	19 (11.7)	92 (7.9)
Dysarthria	307 (45.9)	53 (32.7)	460 (39.5)
Visual field loss	157 (23.5)	29 (17.9)	249 (21.4)
Double vision	36 (5.4)	11 (6.8)	67 (5.8)
Ataxia	93 (13.9)	32 (19.8)	195 (16.8)
Neglect	142 (21.2%)	22 (13.6%)	193 (16.6%)
Sensibility	206 (30.8%)	37 (22.8%)	311 (26.7%)
**No of FAST**,** n (%)**^**§**^			
0 FAST symptoms	104 (15.5)	47 (29.0)	261 (22.4)
1 FAST symptom	184 (27.5)	50 (30.9)	353 (30.3)
2 FAST symptoms	176 (26.3)	35 (21.6)	275 (23.6)
3 FAST symptoms	205 (30.6)	30 (18.5)	275 (23.6)

Percentages in the “Total” column are calculated for all patients (*n* = 1164), whereas subgroup columns include only patients primarily assessed by the EMCC (*n* = 831). The table is presented for descriptive purposes only, and no formal statistical comparisons between groups were performed

IVT: intravenous thrombolysis; EVT: endovascular thrombectomy; NIHSS: National Institutes of Health Stroke Scale; NSR: Norwegian Stroke Registry; FAST: Face – Arm – Speech – Time; EMCC, emergency medical communication centre

^*^ Some variables have patients missing due to incomplete registration in NSR

^**†**^ For ischemic strokes, *n* = 725

^**‡**^ Speaking problem (aphasia) and dysarthria are merged into one variable

^**§**^ The FAST symptoms of speaking problem (aphasia) and dysarthria are merged into one variable

### Factors associated with optimal course

Age and sex were included as adjustment factors. Only “aphasia/dysarthria” showed a statistically significant positive association with the optimal course (Table 2).

**Table 2:** Multivariable logistic regression for association between optimal course and patient- and stroke-related factors and symptoms

Variable	Coefficient	Odds ratio (95% CI)
**Patient- and stroke related factors**		
Age (years)	0.01	1.01 (0.99–1.02)
Sex	0.20	1.22 (0.85–1.76)
Stroke type^**†**^	-0.18	0.82 (0.51–1.35)
Wake-up stroke	0.13	1.13 (0.75–1.71)
**Symptoms**		
Age^**‡**^ (years)	0.02	1.00 (0.99–1.02)
Sex^**‡**^	0.15	1.16 (0.80–1.67)
Vertigo	-0.42	0.66 (0.35–1.25)
Arm paresis	0.02	1.02 (0.64–1.60)
Ataxia	-0.23	0.79 (0.50–1.26)
Double vision	-0.42	0.66 (0.31–1.37)
Facial palsy	0.33	1.39 (0.92–2.09)
Field of vision	0.08	1.08 (0.66–1.78)
Leg paresis	0.10	1.11 (0.70–1.74)
Neglect	0.15	1.16 (0.66–2.04)
Aphasia/dysarthria*	0.42	1.53 (1.03–2.26)
Sensibility	0.24	1.27 (0.82–1.95)

CI: confidence interval

^**†**^ Reference category: ischemic

^**‡**^ Included as adjustment variables

* *p* < 0.05

### Evaluation of the AISMEC model

The confusion matrix for the AI model cohort of 2.980 patients is shown in Table 3.

**Table 3:** Confusion matrix for complete dataset of 2.980 patients

		True values	
		Stroke	No stroke
Predicted values	Stroke	551 (TP)	468 (FP)
	No stroke	133 (FN)	1.828 (TN)
Total		684	2.296

TN: true negative; TP: true positive; FN: false negative; FP: false positive

The diagnostic performance metrics for this cohort are shown in Table 4.

**Table 4:** Diagnostic performance metrics for preliminary testing of the AI model on a complete dataset of 2.980 patients

Metrics	Proportion (%)	95% CI
Sensitivity	80.6	77.6–83.5
Specificity	79.6	78.0–81.3
PPV	54.1	51.0–57.1
NPV	93.2	92.1–94.3
F1-score	64.7	62.0–67.5
Stroke prevalence	23.0	

CI: confidence interval; NPV: Negative predictive value; PPV: Positive predictive value

The AI model achieved a sensitivity of 80.6% (95% CI: 77.6–83.5), specificity of 79.6% (95% CI: 78.0–81.3), PPV of 54.1% (95% CI: 51.0–57.1), NPV of 93.2% (95% CI: 92.1–94.3), and an F1-score of 64.7% (95% CI: 62.0–67.5). PR and ROC curves are presented in Additional file 11.

The model performance and characteristics of the 14 false-negative and 41 false-positive cases are presented in Additional file 9. Within the false-negative group, the model correctly predicted 6 of 14 stroke cases using text classification based on audio only. This number increased to nine when structured clinical data were added. In the false-positive group, the model correctly predicted the absence of stroke in 9 of the 41 cases for both the text-only and combined models (Additional file 10). Due to the small sample sizes, generalizability is limited; therefore, these analyses are exploratory and align with the secondary objective of this study.

## Discussion

We used the AISMEC dataset to characterize stroke patient trajectories through the EMCC, exploring factors associated with an optimal trajectory and whether AI decision support may be less relevant for certain patient groups. We also conducted an exploratory evaluation of the AISMEC AI model, assessing its diagnostic performance on the full dataset and on EMCC stroke false negatives and false positives.

Patient characteristics revealed that fewer patients in the nonoptimal course group received IVT or EVT than those in the optimal course group, underscoring the importance of accurate stroke recognition and triage in EMCC. In contrast, a United States-based study found no significant association between EMCC stroke detection and IVT treatment [25]. In our study, aphasia/dysarthria was more frequently observed in the optimal course group, suggesting that these symptoms may be more readily recognized by callers and EMCC operators. However, a Finnish study reported that speech disturbance was a stroke symptom easily overlooked by EMCC operators [26].

Logistic regression showed that aphasia/dysarthria was the only variable significantly associated with an optimal course through the EMCC. Although there is no causal link to the development of the AI model, this background investigation suggests that the utility of identifying specific symptoms, demographic factors, or stroke–related variables to help determine which patients might benefit from AI-based decision support may be limited. However, the absence of statistically significant associations for other variables should be interpreted with caution. Taken together, these findings suggest potential clinical relevance. AI-based decision support may have the potential to provide benefit for a broad range of stroke-related calls. Therefore, depending on the specific system, it may be relevant to explore the potential role of AI models across all primary medical emergency calls in future studies. However, this study cannot determine whether such a model should also be applied to other non-emergent calls to the EMCC beyond the emergency number-line. This should be considered in the context of local call-handling protocols. These reflections highlight the importance of a thorough understanding of EMCC workflows and how patient groups potentially eligible for AI decision support are currently managed.

To our knowledge, only one comparable AI model has previously been developed [14]. The Danish model relied exclusively on audio in emergency calls and demonstrated a sensitivity of 63.0% and a PPV of 24.9% based on a large dataset comprising over 340,000 incidents. The use of the complete AISMEC model, which uses audio and structured clinical data, may therefore be advantageous, as it appears to perform better than models based on audio analysis alone. However, these findings should be interpreted with caution, as they are based on internal validation and a limited sample size.

We did not compare the model’s performance on the full dataset with that of EMCC operators, as we recently introduced a revised method for calculating EMCC stroke sensitivity [27]. This new approach goes beyond traditional reliance on decision support criterion codes alone, which have traditionally been used for EMCC sensitivity calculations. As the study demonstrated, relying only on these codes may significantly underestimate the true stroke suspicion of EMCC operators. We found a gap in the calculated sensitivity of 63.4% with the traditional method versus 76.8% with the revised approach. The absence of a gold-standard comparator for EMCC performance on the dataset in this study limits the ability to estimate the AI model’s incremental benefit over routine operations. Thus, we will apply this method in future work in the AISMEC project before directly comparing the AISMEC AI model and EMCC operator performance.

Subgroup analyses of FN and FP cases in this study should be interpreted with caution and considered exploratory due to the small sample sizes, and no firm conclusions can be drawn. Numerical interpretation or inference regarding model performance in these subgroups should therefore be avoided. Nevertheless, AI decision support may be particularly relevant for EMCC FN cases of stroke, as it may help identify patients who might otherwise go undetected and miss treatment opportunities. In such cases, the potential benefits for patients and society are substantial. It is difficult to define a specific threshold for when AI support becomes cost-effective, but the number needed to treat to achieve a one-level improvement in outcome according to the modified Rankin Scale is 2.6 for EVT and 3.1 for IVT (administered within 90 min), indicating that timely treatment with IVT and/or EVT is highly desirable [28, 29].

For the EMCC stroke false-positive subgroup, the benefits of improved accuracy are primarily system- and resource-related. The use of AI decision support to rule out stroke as a likely diagnosis, thereby reducing unnecessary dispatch of ambulance resources, would be highly desirable from a system perspective. A Norwegian study reported a PPV for stroke of 16% at the Oslo EMCC [30], leading to a high number of potentially unnecessary ambulance dispatches. The exploratory evaluation in this study may suggest that the AISMEC AI model can leverage both audio logs and structured clinical data to provide decision support for these clinically important groups [31].

Operational challenges must also be considered. As highlighted in a Danish study on AI decision support for out-of-hospital cardiac arrest, these include integration with existing systems, limited data capacity, and operator trust and training requirements [32]. In addition, external validation in independent datasets and prospective evaluation are required before the model can be considered for clinical implementation. Ensuring the sustainable implementation and use of AI decision support in EMCCs will be crucial in the coming stages, and the use of international consensus guidelines is advised [33].

### Limitations

Several limitations should be considered. The study’s operational definition of a “nonoptimal course” (based on a 24-h cutoff and acute priority dispatch criteria) is restrictive and may not capture all clinically relevant cases of suboptimal care. Conversely, it could classify some borderline cases as nonoptimal. This creates a potential for under- or over-classification; however, it was created based on clinical considerations and the lack of standardized definitions. Patient delay can clearly also interfere with the time from ictus to first contact. Taken together, misclassification represents a limitation and may have influenced the analyses in the study. In addition, alternative definitions of patient trajectories could be considered, as different thresholds may have resulted in different classifications, particularly with respect to the time-critical nature of acute stroke treatment. A formal sensitivity analysis was not performed and should be addressed in future studies.

In addition, the AI model was trained and evaluated using data from both a case-enriched subset and a randomly selected subset of the cohort, which limits the generalisability of the results and may inflate prevalence-dependent performance metrics, particularly the positive predictive value. The model performance reflects internal validation only and should be interpreted accordingly. Therefore, the reported performance metrics may not fully reflect the model’s accuracy when applied to a broader EMCC population. Further testing using larger and more representative datasets will be conducted within the AISMEC project, which may reduce bias toward a high positive predictive value caused by the overrepresentation of stroke patients in our cohort. In addition, performance metrics that depend on disease prevalence, such as positive predictive value (PPV) and F1-score, may be overestimated compared with values expected under true population prevalence.

A comparison with human operator performance for stroke assessment is currently lacking and will be addressed in continued model development within AISMEC and presented in a subsequent study. The incremental value of the AI model compared with current EMCC practice therefore remains unknown.

Furthermore, the number of patients included in the EMCC false-negative (*n* = 14) and false-positive (*n* = 41) subgroups was limited. The evaluation of these subgroups is exploratory and hypothesis-generating and should be interpreted with caution. The identification of false-negative and false-positive cases was conducted by a single author (EI), which may introduce classification bias. This process should involve multiple reviewers in future studies.

Information on whether the caller was the patient or a third party was not consistently available. This may have influenced the communication and identification of symptoms requiring verbal interaction, such as dysarthria and aphasia, as these rely on both EMCC operator-observed speech disturbances when the patient is the caller and on reported symptoms when a third party initiates the call.

Another limitation is that laboratory variables indicative of stroke risk (e.g., cholesterol and glucose levels) were not available for patients from 2019 and were therefore not included in the initial model development. This may have limited the model’s ability to capture certain aspects of vascular risk and may have influenced overall model performance. The inclusion of laboratory data is planned in future iterations of the AISMEC AI model.

The system-specific operational characteristics of EMCCs in Norway poses challenges for cross-study comparability. However, since a similar framework is used (criteria-based decision support combined with EMCC operator-driven evaluation and dispatch), we believe it would be feasible to conduct similar studies in other EMCC environments.

Finally, the retrospective study design limits the extent to which real-time use of the AI model can be explored. As this was an exploratory evaluation, a prospective implementation study was beyond the scope of the present work.

## Conclusions

This exploratory evaluation indicates the potential of a novel multimodal AI pipeline to support stroke detection in medical emergency calls. Combining audio analysis with structured clinical data appears feasible and internal evaluation yields promising results. Further research using larger and more diverse datasets is warranted to validate these findings and assess their applicability in real‑world settings.

## Supplementary Information

Below is the link to the electronic supplementary material.

Supplementary Material 1: File name: Additional file 1: Index page: Stroke protocol. File format: PNG. Title of data: Index page – Stroke protocol. Description of data: Screenshot of Index page 27 stroke symptoms, showing the protocol with criteria for the Emergency Medical Communication Centre (EMCC) operator and the three urgency levels: “acute” (ambulance with lights and sirens), “urgent” (ambulance dispatched or call transferred to Out-of-Hours Primary Care Centre), and “regular” (non-urgent).

Supplementary Material 2: File name: Additional file 2: Variables from the Norwegian Stroke Registry (NSR). File format: Word (.docx). Title of data: Variables from Norwegian Stroke Registry (NSR). Description of data: From the NSR, we obtained data registered by the hospital for each patient after stroke.

Supplementary Material 3: File name: Additional file 3: Variables from the Emergency Medical Communication Centre (EMCC; Emergency Medical Information System [AMIS]). File format: Word (.docx). Title of data: Variables from the Emergency Medical Communication Centre (EMCC; Emergency Medical Information System [AMIS]). Description of data: From the EMCC patient record system in Bergen, AMIS, using the patients’ social security number, we extracted the patients’ prehospital contact points and measures taken by the Emergency Medical Services.

Supplementary Material 4: File name: Additional file 4: Variables from the Norwegian Prescribed Drug Registry. File format: Word (.docx). Title of data: Variables from the Norwegian Prescribed Drug Registry. Description of data: From Norwegian Prescribed Drug Registry, we obtained data on prescribed medications relevant to stroke risk. For each patient, we checked the use of medication with ATC-codes evaluated by clinicians in the research group (HIH, EI) to be associated with an increased stroke risk.

Supplementary Material 5: File name: Additional file 5: Variables from hospital records (DIPS). File format: Word (.docx). Title of data: Variables from hospital records (DIPS). Description of data: Variables collected for each patient, if a prior history is recorded at the hospital, from hospital records.

Supplementary Material 6: File name: Additional file 6: Description of artificial intelligence (AI) model development. File format: Word (.docx). Title of data: Description of artificial intelligence (AI) model development. Description of data: Detailed description of the two pipelines (Audio pipeline and Structured clinical data pipeline) combined to create the Hybrid Model Pipeline, providing the final probability estimate of stroke.

Supplementary Material 7: File name: Additional file 7: Flowchart of trajectory cohort. File format: Word (.docx) Title of data: Flowchart of trajectory cohort. Description of data: Flowchart showing selection of trajectory cohort.

Supplementary Material 8: File name: Additional file 8: Flowchart of artificial intelligence (AI) model population with stroke false positive and false negative cases. File format: Word (.docx). Title of data: Flowchart of artificial intelligence (AI) model population with stroke false positive and false negative cases. Description of data: Flowchart showing selection of AI model cohort with stroke false positive and false negatives.

Supplementary Material 9: File name: Additional file 9: Characteristics and artificial intelligence (AI) model performance for false negative and false positive cases. File format: Word (.docx). Title of data: Characteristics and artificial intelligence (AI) model performance for false negative and false positive cases. Description of data: Table presenting age, sex, and if the patients have relevant stroke risk hospital history or prescribed medications, as well as results of AMI model prediction for stroke based on audio only and audio combined with hospital history and medication.

Supplementary Material 10: File name: Additional file 10: Detailed artificial intelligence (AI) model results for stroke false negative and false positive cases. File format: Excel (.xls). Title of data: Detailed artificial intelligence (AI) model results for stroke false negative and false positive cases. Description of data: Two Excel sheets (stroke false negative and false positive cases) showing details of the AI model´s performance on stroke prediction for the two subgroups. Variables shown in the file are: age, sex, hospital history, has prescribed medications, model prediction audio log, correct prediction audio log, prediction combined, correct prediction combined.

Supplementary Material 11: File name: Additional file 11: Precision–recall and receiver operating characteristics curves for AI model performance on AI model cohort of 2980 patients. File format: Word (.docx)Title of data: Precision–recall and receiver operating characteristics curves for AI model performance on AI model cohort of 2980 patients. Description of data: Two figures (PR and ROC curves) for AI model testing on 2980 patients. Each figure has three lines, one for each part of the three-fold stratified cross-validation.

## Data Availability

The datasets generated and analyzed during the current study are not publicly available due to legal regulations concerning sensitive health data. A deidentified data set, data dictionary, and analytic code for this investigation is available on reasonable request, from the date of publication. Please contact Emil Iversen, MD, at email [emil.iversen@helse-bergen.no].

## Abbreviations

AI: Artificial intelligence
CI: Confidence interval
EMCCs: Emergency medical communication centres
EVT: Endovascular thrombectomy
FAST: Face-arm-speech-time
FN: False negative
FP: False positive
IVT: Intravenous thrombolysis
LR: Logistic regression
NSR: Norwegian stroke registry
NPV: Negative predictive value
OR: Odds ratio
PR: Precision–recall
PPV: Positive predictive value
ROC: Receiver operating characteristics
STROBE: Strengthening the reporting of observational studies in epidemiology
TN: True negative
TP: True positive

## References

[1] Wenstrup J, Hestoy BH, Sagar MV, Blomberg SNF, Christensen H, Christensen HC, et al. Emergency Medical Services dispatcher recognition of stroke: a systematic review. Eur Stroke J. 2024;9:283–94. 10.1177/23969873231223339. 38174575 10.1177/23969873231223339 PMC11318428

[2] Meretoja A, Keshtkaran M, Tatlisumak T, Donnan GA, Churilov L. Endovascular therapy for ischemic stroke: Save a minute-save a week. Neurology. 2017;88:2123–7. 10.1212/wnl.0000000000003981. 28455382 10.1212/WNL.0000000000003981

[3] Powers WJ, Rabinstein AA, Ackerson T, Adeoye OM, Bambakidis NC, Becker K, et al. Guidelines for the early management of patients with acute ischemic stroke: 2019 update to the 2018 Guidelines for the early management of acute ischemic stroke: a guideline for healthcare professionals from the American Heart Association/American Stroke Association. Stroke. 2019;50:e344–418. 10.1161/str.0000000000000211. 31662037 10.1161/STR.0000000000000211

[4] Berglund A, Svensson L, Sjöstrand C, von Arbin M, von Euler M, Wahlgren N, et al. Higher prehospital priority level of stroke improves thrombolysis frequency and time to stroke unit: the Hyper Acute Stroke Alarm (HASTA) study. Stroke. 2012;43:2666–70. 10.1161/strokeaha.112.652644. 22879096 10.1161/STROKEAHA.112.652644

[5] Ekundayo OJ, Saver JL, Fonarow GC, Schwamm LH, Xian Y, Zhao X, et al. Patterns of emergency medical services use and its association with timely stroke treatment: findings from Get With the Guidelines-Stroke. Circ Cardiovasc Qual Outcomes. 2013;6:262–9. 10.1161/circoutcomes.113.000089. 23633218 10.1161/CIRCOUTCOMES.113.000089

[6] Caceres JA, Adil MM, Jadhav V, Chaudhry SA, Pawar S, Rodriguez GJ, et al. Diagnosis of stroke by emergency medical dispatchers and its impact on the prehospital care of patients. J Stroke Cerebrovasc Dis. 2013;22:e610–4. 10.1016/j.jstrokecerebrovasdis.2013.07.039. 24075587 10.1016/j.jstrokecerebrovasdis.2013.07.039

[7] Eliakundu AL, Cadilhac DA, Kim J, Kilkenny MF, Bagot KL, Andrew E, et al. Determining the sensitivity of emergency dispatcher and paramedic diagnosis of stroke: statewide registry linkage study. J Am Coll Emerg Physicians Open. 2022;3:e12750. 10.1002/emp2.12750. 35795711 10.1002/emp2.12750 PMC9249375

[8] The Norwegian Directorate of Health. The norwegian index for emergency medical assistance. 2018.

[9] Ellensen EN, Naess H, Wisborg T, Hunskaar S, Zakariassen E. Stroke identification by criteria based dispatch - a register based study. Acta Anaesthesiol Scand. 2018;62:105–15. 10.1111/aas.13032. 29105736 10.1111/aas.13032 PMC5725681

[10] Duvekot MHC, Kerkhoff H, Venema E, Bos HWDJC, Smeekes D, Buijck BI, et al. Medical attention seeking by suspected stroke patients: emergency medical services or general practitioner? Clin Neurol Neurosurg. 2022;218:107297. 10.1016/j.clineuro.2022.107297. 35636379 10.1016/j.clineuro.2022.107297

[11] Faiz KW, Sundseth A, Thommessen B, Rønning OM. Prehospital delay in acute stroke and TIA. Emerg Med J. 2013;30:669–74. 10.1136/emermed-2012-201543. 22886891 10.1136/emermed-2012-201543

[12] Doggen CJ, Zwerink M, Droste HM, Brouwers PJ, van Houwelingen GK, van Eenennaam FL, et al. Prehospital paths and hospital arrival time of patients with acute coronary syndrome or stroke, a prospective observational study. BMC Emerg Med. 2016;16:3. 10.1186/s12873-015-0065-y. 26748628 10.1186/s12873-015-0065-y PMC4706997

[13] Jamtli B, Svendsen EJ, Jørgensen TM, Kramer-Johansen J, Hov MR, Hardeland C. Factors affecting emergency medical dispatchers decision making in stroke calls - a qualitative study. BMC Emerg Med. 2024;24:214. 10.1186/s12873-024-01129-0. 39548378 10.1186/s12873-024-01129-0 PMC11566283

[14] Wenstrup J, Havtorn JD, Borgholt L, Blomberg SN, Maaloe L, Sayre MR, et al. A retrospective study on machine learning-assisted stroke recognition for medical helpline calls. NPJ Digit Med. 2023;6:235. 10.1038/s41746-023-00980-y. 38114611 10.1038/s41746-023-00980-y PMC10730829

[15] Nicoletta V, Robitaille-Fortin M, Bélanger V, Mercier É, Harrisson J. Performance measures of the medical priority dispatch system in an urban basic life support system. Scand J Trauma Resusc Emerg Med. 2025;33:94. 10.1186/s13049-025-01410-6. 40399996 10.1186/s13049-025-01410-6 PMC12096499

[16] Scholz ML, Collatz-Christensen H, Blomberg SNF, Boebel S, Verhoeven J, Krafft T. Artificial intelligence in Emergency Medical Services dispatching: assessing the potential impact of an automatic speech recognition software on stroke detection taking the Capital Region of Denmark as case in point. Scand J Trauma Resusc Emerg Med. 2022;30:36. 10.1186/s13049-022-01020-6. 35549978 10.1186/s13049-022-01020-6 PMC9097123

[17] Rudd AG, Bladin C, Carli P, De Silva DA, Field TS, Jauch EC, et al. Utstein recommendation for emergency stroke care. Int J Stroke. 2020;15:555–64. 10.1177/1747493020915135. 32223543 10.1177/1747493020915135 PMC7672780

[18] Faiz KW, Sundseth A, Thommessen B, Rønning OM. Prehospital path in acute stroke. Tidsskr Nor Laegeforen. 2017;137:798–802. 10.4045/tidsskr.16.0512. 28597634 10.4045/tidsskr.16.0512

[19] Registry TNS. Acute form. The Norwegian Stroke Registry. 2018/2019.

[20] Brott T, Adams HP Jr., Olinger CP, Marler JR, Barsan WG, Biller J, et al. Measurements of acute cerebral infarction: a clinical examination scale. Stroke. 1989;20:864–70. 10.1161/01.str.20.7.864. 2749846 10.1161/01.str.20.7.864

[21] Radford A, Kim JW, Xu T, Brockman G, Mcleavey C, Sutskever. I. Robust speech recognition via large-scale weak supervision. International Conference on Machine Learning. 2023:28492-518.

[22] Warner B, Chaffin A, Clavié B, Weller O, Hallström O, Taghadouini S, et al. Smarter, better, faster, longer: A modern bidirectional encoder for fast, memory efficient, and long context finetuning and inference. arXiv preprint arXiv. 2024;1:241213663.

[23] Breiman L. Random Forests. Mach Learn. 2001;45:5–32. 10.1023/A:1010933404324.

[24] von Elm E, Altman DG, Egger M, Pocock SJ, Gøtzsche PC, Vandenbroucke JP, et al. Strengthening the Reporting of Observational Studies in Epidemiology (STROBE) statement: guidelines for reporting observational studies. BMJ. 2007;335:806–8. 10.1136/bmj.39335.541782.AD. 17947786 10.1136/bmj.39335.541782.AD PMC2034723

[25] Oostema JA, Chassee T, Reeves M. Emergency dispatcher stroke recognition: associations with downstream care, Prehospital emergency care: official journal of the national association of EMS physicians and the national association of state EMS directors. Prehosp Emerg Care. 2018. 10.1080/10903127.2017.1405131

[26] Mattila OS, Puolakka T, Ritvonen J, Pihlasviita S, Harve H, Alanen A, et al. Targets for improving dispatcher identification of acute stroke. Int J Stroke. 2019;14:409–16. 10.1177/1747493019830315. 30758276 10.1177/1747493019830315

[27] Iversen E, Ihle-Hansen H, Halle KK, Lundervold AS, Myrmel L, Vestbø AS, et al. Stroke sensitivity calculation in medical emergency calls and factors associated with stroke suspicion: a retrospective registry-based study. Ann Emerg Med. 2025;86:533–40. 10.1016/j.annemergmed.2025.04.028. 40448981 10.1016/j.annemergmed.2025.04.028

[28] Goyal M, Menon BK, van Zwam WH, Dippel DW, Mitchell PJ, Demchuk AM, et al. Endovascular thrombectomy after large-vessel ischaemic stroke: a meta-analysis of individual patient data from five randomised trials. Lancet. 2016;387:1723–31. 10.1016/S0140-6736(16)00163-X. 26898852 10.1016/S0140-6736(16)00163-X

[29] Saver JL. Number needed to treat estimates incorporating effects over theentire range of clinical outcomes: Novel derivation method and application to thrombolytic therapy for acute ttroke. Arch Neurol. 2004;61:1066–70. 10.1001/archneur.61.7.1066. 15262737 10.1001/archneur.61.7.1066

[30] Jamtli B, Hov MR, Jorgensen TM, Kramer-Johansen J, Ihle-Hansen H, Sandset EC, et al. Telephone triage and dispatch of ambulances to patients with suspected and verified acute stroke - a descriptive study. BMC Emerg Med. 2024;24:43. 10.1186/s12873-024-00962-7. 38486156 10.1186/s12873-024-00962-7 PMC10941420

[31] Jackson GP, Shortliffe EH. Understanding the evidence for artificial intelligence in healthcare. BMJ Qual Saf. 2025;34:421–4. 10.1136/bmjqs-2025-018559. 40246317 10.1136/bmjqs-2025-018559 PMC12229047

[32] Blomberg SN, Christensen HC, Lippert F, Ersbøll AK, Torp-Petersen C, Sayre MR, et al. Effect of machine learning on dispatcher recognition of out-of-hospital cardiac arrest during calls to emergency medical services: a randomized clinical trial. JAMA Netw Open. 2021;4:e2032320. 10.1001/jamanetworkopen.2020.32320. 33404620 10.1001/jamanetworkopen.2020.32320 PMC7788469

[33] Lekadir K, Frangi AF, Porras AR, Glocker B, Cintas C, Langlotz CP, et al. FUTURE-AI: international consensus guideline for trustworthy and deployable artificial intelligence in healthcare. BMJ. 2025;388:e081554. 10.1136/bmj-2024-081554. 39909534 10.1136/bmj-2024-081554 PMC11795397

