# Supplementary material for: Stroke detection in medical emergency calls: a retrospective analysis and exploratory evaluation of an AI decision support model
Source: Scand J Trauma Resusc Emerg Med. 2026 Jun 9;34:138. doi: 10.1186/s13049-026-01645-x (PMC13487976; doi:10.1186/s13049-026-01645-x)
Supplement: Supplementary file 1 [file 13049_2026_1645_MOESM11_ESM.docx]

**Additional file 11:** Precision–recall and receiver operating characteristics curve for AI model performance on AI model cohort of 2980
