# Supplementary material for: Stroke detection in medical emergency calls: a retrospective analysis and exploratory evaluation of an AI decision support model
Source: Scand J Trauma Resusc Emerg Med. 2026 Jun 9;34:138. doi: 10.1186/s13049-026-01645-x (PMC13487976; doi:10.1186/s13049-026-01645-x)
Supplement: Supplementary file 2 [file 13049_2026_1645_MOESM2_ESM.docx]

**Additional file 2: Variables from The Norwegian Stroke Registry (NSR)**

From the NSR, we obtained data registered by the hospital for each patient after stroke. The following variables were provided:

- symptoms
- National Institutes of Health Stroke Scale (NIHSS) score upon arrival and pre-/post-intravenous thrombolysis (IVT)
- type of stroke (ischemic or bleeding)
- sex
- age
- wake-up stroke
- treatment with IVT or endovascular thrombectomy

The symptom variable ”Level of consciousness (upon hospital admission)“ was dichotomized into “Awake” (0) and “Others” (1), with “Others” encompassing all other levels of consciousness (1 = Drowsy, responds adequately to light stimulation; 2 = Drowsy, reacts only to stronger/repeated stimulation; and 3 = Does not respond, or only with non-targeted movement).
