# Supplementary material for: Stroke detection in medical emergency calls: a retrospective analysis and exploratory evaluation of an AI decision support model
Source: Scand J Trauma Resusc Emerg Med. 2026 Jun 9;34:138. doi: 10.1186/s13049-026-01645-x (PMC13487976; doi:10.1186/s13049-026-01645-x)
Supplement: Supplementary file 3 [file 13049_2026_1645_MOESM3_ESM.docx]

**Additional file 3: Variables from the Emergency Medical Communication Center (EMCC; Emergency Medical Information System [AMIS])**

From the EMCC patient record system in Bergen, Emergency Medical Information System, using the patients’ social security number, we extracted the patients’ prehospital contact points and measures taken by the Emergency Medical Services. This included the following:

- The time of stroke onset
- All telephone communication from and about the patient
- Assumed contact between patient and relatives/others
- Dispatch of car ambulance/helicopter/boat ambulance/primary care doctor/home nursing care
- Free text written by the EMCC operator
- Degree of urgency set by the EMCC operator
- Telephone line into Bergen EMCC
- Other actions

Every event was registered with a timestamp, resulting in a detailed chronological mapping of every patient´s journey until arrival at the hospital. In cases where the prehospital contact points were unclear, we consulted the emergency call audio logs to complete the mapping (for 2019 patients only, due to the lack of audio logs for 2018).
