# Supplementary material for: Stroke detection in medical emergency calls: a retrospective analysis and exploratory evaluation of an AI decision support model
Source: Scand J Trauma Resusc Emerg Med. 2026 Jun 9;34:138. doi: 10.1186/s13049-026-01645-x (PMC13487976; doi:10.1186/s13049-026-01645-x)
Supplement: Supplementary file 4 [file 13049_2026_1645_MOESM4_ESM.docx]

**Additional File 4: Variables from the Norwegian Prescribed Drug Registry**

From the Norwegian Prescribed Drug Registry, we obtained data on prescribed medications relevant to stroke risk and prescription dates. For each patient, we checked for the use of medication with the following ATC codes (specified when only certain subgroups were obtained):

- A A[limentary tract and metabolism](https://atcddd.fhi.no/atc_ddd_index/?code=A&showdescription=no)
  - A08 A[ntiobesity preparations, excl. diet products](https://atcddd.fhi.no/atc_ddd_index/?code=A08&showdescription=no)
  - A10 D[rugs used in diabetes](https://atcddd.fhi.no/atc_ddd_index/?code=A10&showdescription=no)
- B [blood and blood forming organs](https://atcddd.fhi.no/atc_ddd_index/?code=B&showdescription=no)
  - B01 A[ntithrombotic agents](https://atcddd.fhi.no/atc_ddd_index/?code=B01&showdescription=no)
  - B02 A[ntihemorrhagics](https://atcddd.fhi.no/atc_ddd_index/?code=B02&showdescription=no)
- C C[ardiovascular system](https://atcddd.fhi.no/atc_ddd_index/?code=C&showdescription=yes)
- M M[usculo-skeletal system](https://atcddd.fhi.no/atc_ddd_index/?code=M&showdescription=yes)
  - M01 [antiinflammatory and antirheumatic products](https://atcddd.fhi.no/atc_ddd_index/?code=M01&showdescription=no)
    - M01a A[ntiinflammatory and antirheumatic products, non-steroids](https://atcddd.fhi.no/atc_ddd_index/?code=M01A&showdescription=no)
- N N[ervous system](https://atcddd.fhi.no/atc_ddd_index/?code=N&showdescription=yes)
  - N02 A[nalgesics](https://atcddd.fhi.no/atc_ddd_index/?code=N02&showdescription=no)
    - N02B O[ther analgesics and antipyretics](https://atcddd.fhi.no/atc_ddd_index/?code=N02B&showdescription=no)
      - N02BA S[alicylic acid and derivatives](https://atcddd.fhi.no/atc_ddd_index/?code=N02BA&showdescription=no)
- R R[espiratory system](https://atcddd.fhi.no/atc_ddd_index/?code=R&showdescription=yes)
  - R03 D[rugs for obstructive airway diseases](https://atcddd.fhi.no/atc_ddd_index/?code=R03&showdescription=no)
