# Supplementary material for: Stroke detection in medical emergency calls: a retrospective analysis and exploratory evaluation of an AI decision support model
Source: Scand J Trauma Resusc Emerg Med. 2026 Jun 9;34:138. doi: 10.1186/s13049-026-01645-x (PMC13487976; doi:10.1186/s13049-026-01645-x)
Supplement: Supplementary file 5 [file 13049_2026_1645_MOESM5_ESM.docx]

**Additional File 5: Variables from hospital records (DIPS)**

From the hospital records of each patient, we obtained data relevant to stroke risk. The following variables were obtained from previous contacts with the hospital:

- Time of admission
- Clinic/ward
- Elective or Emergency
- Length of stay
- Diagnoses (ICD10)
- Treatments (procedures)
