# Supplementary material for: Stroke detection in medical emergency calls: a retrospective analysis and exploratory evaluation of an AI decision support model
Source: Scand J Trauma Resusc Emerg Med. 2026 Jun 9;34:138. doi: 10.1186/s13049-026-01645-x (PMC13487976; doi:10.1186/s13049-026-01645-x)
Supplement: Supplementary file 6 [file 13049_2026_1645_MOESM6_ESM.docx]

**Additional File 6: Description of artificial intelligence (AI) model development**

This section details the development and implementation of a pipeline designed to predict the likelihood of stroke based on an analysis of emergency call audio and associated structured clinical data. The pipeline leverages recent advances in Automatic Speech Recognition (ASR) and Natural Language Processing (NLP) to achieve a robust and informative prediction system. The core of the pipeline consists of a fine-tuned transcription model and a subsequent classification model, supplemented by a parallel classification model built on structured clinical data.

## Audio pipeline

### *Transcription model fine-tuning*

Accurate transcription of emergency call audio is a critical first step in identifying potential patients with stroke. Pre-trained ASR models offer general transcription capabilities; however, their performance often suffers when applied to specific domains, such as emergency calls, which are characterized by diverse accents, background noise, and specialized vocabulary. To address this, we fine-tuned a Whisper model, a state-of-the-art ASR system, specifically for emergency call data originating from the Vestland Region, Norway (Bergen).

The fine-tuning process was conducted using a dataset of 86 manually transcribed audio files. The process used the same general preprocessing and feature handling approach used for fine-tuning Nb-Whisper. The same optimisation technique was used as well as training hyperparameters. The audio files were meticulously annotated by human experts to ensure high fidelity and accuracy. The dataset was partitioned into three subsets: 80% for training (n=69), 10% for validation, and 10% for testing. We initialized the Whisper model from the nb-whisper checkpoint, a variant pre-trained on a substantial corpus of Norwegian-language data, including various regional dialects. This provided a strong foundation for adapting the model to the specific characteristics of emergency call audio.

We fine-tuned the model with half-precision (fp16), using a ${10}^{-5}$, 40 maximum steps with 64 batch size, gradient checkpointing and accumulation steps, evaluation every 5 steps.

### Following fine-tuning, the model performance was evaluated using the Word Error Rate (WER) as the primary metric. The original nb-whisper model achieved a WER of 0.83 on the test dataset. Our fine-tuned model demonstrated a 5% relative improvement, achieving a WER of 0.78 for the test set. This reduction in the WER indicates a significant enhancement in transcription accuracy, which is crucial for downstream NLP tasks.

### *Stroke prediction classification model*

The transcribed text from the emergency calls served as input to a classification model designed to predict the probability of a patient experiencing a stroke. We selected the ModernBERT architecture for this task because of its ability to effectively process long sequences. Emergency call transcriptions can vary considerably in length, often exceeding the context window limitations of standard BERT-based models. ModernBERT’s extended context length of 8192 tokens allows for the incorporation of complete call transcripts without truncation, preserving potentially valuable information for training. We followed the general training set-up of the original ModernBERT, i.e., next token prediction with cross-entropy loss.

The overall pipeline structure is as follows: emergency call audio → Whisper → transcription → ModernBERT → classification. The output of the ModernBERT model was a stroke probability score ranging from 0 to 1, with higher values indicating a greater likelihood of stroke.

The classification model was fine-tuned on a dataset of 1708 emergency call transcriptions, partitioned similarly to the transcription dataset: 80%, 10%, and 10% for training, validation, and testing, respectively. The dataset used for training was balanced, meaning there were equal numbers of elements with classification of strokes and no strokes. The fine-tuning was carried out with 500 maximum steps, a batch size of 128 with gradient checkpointing and accumulation steps, and evaluation every 50 steps.

The resulting fine-tuned classifier achieved an F1-score of 0.8 on the independent test dataset. The precision was slightly higher than the recall, at 82% and 78%, respectively.

## Structured data pipeline

A separate classification model was developed for the structured clinical data, which consisted of historical patient information from hospital records, including demographics, pre-existing conditions, and medication prescriptions. A Random Forest algorithm was selected because of its robustness to noisy data and ability to model complex, nonlinear relationships. To account for the class imbalance present in the dataset, the Balanced Random Forest algorithm was applied [1]. Prior to model training, categorical variables were encoded using a one-hot encoding scheme.

Model selection and parameter tuning were performed using a three-fold stratified cross-validation procedure, ensuring that each fold preserved the proportions of stroke and non-stroke cases. This approach allowed for a robust estimation of the model performance and informed the final choice of parameters.

Table showing features included in the model using structured data

## Hybrid model pipeline

The two pipelines developed for audio and structured data were integrated to produce a final probability estimate for stroke. Several strategies for model fusion were evaluated, and the best performance was obtained when the probability output from the ModernBERT-based transcription classifier was incorporated as an additional input feature in the structured data classification model. This hybrid approach leverages complementary information from both unstructured and structured sources, thereby enhancing predictive accuracy compared to using either data type alone.

**References**

1.	Chen C, Breiman L. Using random forest to learn imbalanced data. University of California, Berkeley, 2004.
