# Supplementary material for: Stroke detection in medical emergency calls: a retrospective analysis and exploratory evaluation of an AI decision support model
Source: Scand J Trauma Resusc Emerg Med. 2026 Jun 9;34:138. doi: 10.1186/s13049-026-01645-x (PMC13487976; doi:10.1186/s13049-026-01645-x)
Supplement: Supplementary file 7 [file 13049_2026_1645_MOESM7_ESM.docx]

**Additional file 7:** Flowchart of trajectory cohort

NSR: Norwegian Stroke Registry, EMCC: Emergency Medical Communication Center

*Patients with in-hospital stroke or transferred from another hospital region or abroad.
