# Supplementary material for: Stroke detection in medical emergency calls: a retrospective analysis and exploratory evaluation of an AI decision support model
Source: Scand J Trauma Resusc Emerg Med. 2026 Jun 9;34:138. doi: 10.1186/s13049-026-01645-x (PMC13487976; doi:10.1186/s13049-026-01645-x)
Supplement: Supplementary file 8 [file 13049_2026_1645_MOESM8_ESM.docx]

**Additional file 8:** Flowchart of artificial intelligence (AI) model population with stroke false positives and false negatives

EMCC: Emergency Medical Communication Center

*Based on manual review as described in the EMCC false negative and false positive subgroups-section
