# Supplementary material for: Stroke detection in medical emergency calls: a retrospective analysis and exploratory evaluation of an AI decision support model
Source: Scand J Trauma Resusc Emerg Med. 2026 Jun 9;34:138. doi: 10.1186/s13049-026-01645-x (PMC13487976; doi:10.1186/s13049-026-01645-x)
Supplement: Supplementary file 9 [file 13049_2026_1645_MOESM9_ESM.docx]

**Additional file 9: Characteristics and AI model performance for false-negative and false-positive cases**

|  | **False-negative cases (n=14)** | **False-positive cases (n=41)** |
| --- | --- | --- |
| Age in years, median (Q1–Q3) | 75 (69–87) | 78 |
| Sex (women) | 6 | 23 |
| Has hospital history | 7 | 30 |
| Has prescribed medications | 10 | 39 |
| Correct model prediction on audio log | 6 | 9 |
| Correct model prediction on combined data | 9 | 9 |

AI: artificial intelligence
